# Assessment of the clinical and analytical performance of three Seegene Allplex SARS-CoV-2 assays within the VALCOR framework

**DOI:** 10.1128/spectrum.02397-23

**Published:** 2024-01-08

**Authors:** Pui Yan Jenny Chung, Sharonjit K. Dhillon, Cindy Simoens, Lize Cuypers, Lies Laenen, Jesper Bonde, Philippe Corbisier, Gerhard Buttinger, Clementina E. Cocuzza, Steven Van Gucht, Marc Van Ranst, Marc Arbyn

**Affiliations:** 1 Unit of Cancer Epidemiology, Belgian Cancer Centre, Sciensano, Brussels, Belgium; 2 Department of Laboratory Medicine, National Reference Centre for Respiratory Pathogens, University Hospitals Leuven, Leuven, Belgium; 3 Laboratory of Clinical Microbiology, Department of Microbiology, Immunology and Transplantation, KU Leuven, Leuven, Belgium; 4 Molecular Pathology Laboratory, Department of Pathology, AHH-Hvidovre Hospital, Copenhagen University Hospital, Copenhagen, Denmark; 5 Joint Research Centre (JRC), European Commission, Geel, Belgium; 6 Laboratory of Clinical Microbiology and Virology, Department of Medicine and Surgery, University of Milano-Bicocca, Monza, Italy; 7 Service of Viral Diseases, Sciensano, Brussels, Belgium; 8 Laboratory of Clinical and Epidemiological Virology, Department of Microbiology, Immunology and Transplantation, Rega Institute for Medical Research, KU Leuven, Leuven, Belgium; 9 Department of Human Structure and Repair, Faculty of Medicine and Health Sciences, University of Ghent, Ghent, Belgium; National Institute of Allergy and Infectious Diseases, Baltimore, Maryland, USA

**Keywords:** SARS-CoV-2, diagnostics test accuracy, RT-PCR, COVID-19, test validation, quality control, Seegene, Allplex, VALCOR, standard, reference material

## Abstract

The coronavirus disease 2019 (COVID-19) pandemic demonstrated the need for accurate diagnostic testing for the early detection of severe acute respiratory syndrome coronavirus 2 (SARS-CoV-2). Although the pandemic has ended, accurate assays are still needed to monitor viral spread at national levels and beyond through population and wastewater surveillance. To enhance early detection, SARS-CoV-2 assays should have high diagnostic accuracy and should be validated to assure accurate results. Three distinct SARS-CoV-2 assays were evaluated with clinical samples using the VALCOR (VALidation of SARS-CORona Virus-2 assays) framework, with the TaqPath COVID-19 assay (ThermoFisher Scientific, USA) as a comparator. We evaluated clinical sensitivity, specificity, limit of detection (LOD), and overall concordance between comparator and three index Allplex SARS-CoV-2 assays (Seegene, South Korea): Allplex-SC2, Allplex-SC2Fast (Fast PCR), and Allplex-SC2FabR (SARS-CoV-2/FluA/FluB/respiratory syncytial virus). Analytical performance and LOD of index assays were assessed using a dilution series of three synthetic SARS-CoV-2 sequence reference materials (RMs). Ninety SARS-CoV-2 positives and 90 SARS-CoV-2 negatives were tested. All Allplex assays had 100.0% sensitivity (95%CI = 95.9%–100.0%). Allplex-SC2 and Allplex-SC2Fast assays had 97.8% specificity (95%CI = 92.3%–99.7%) and 98.9% overall concordance [*κ* = 0.978 (95%CI = 0.947–1.000)]. Allplex-SC2FabR assay showed 100.0% specificity (95%CI = 95.9%–100.0%) and 100.0% overall concordance [*κ* = 1.000 (95%CI = 1.000–1.000)]. LOD assessment of index assays revealed detection down to 2.61 × 10^2^ copies/mL in clinical samples, while the analytical LOD was 9.00 × 10^2^ copies/mL. In conclusion, the evaluation of the three Seegene Allplex SARS-CoV-2 assays showed high sensitivity and specificity and an overall good assay concordance with the comparator. The assays showed low analytical LOD using RM and even a slightly lower LOD in clinical samples. Non-overlapping target gene sequences between SARS-CoV-2 assays and RMs emphasize the need for aligning targeted sequences of diagnostic assays and RMs.

**IMPORTANCE:**

The coronavirus disease 2019 pandemic has a significant impact on global public health, economies, and societies. As shown through the first phases of the pandemic, accurate and timely diagnosis is crucial for disease control, prevention, and monitoring. Though the pandemic phase of severe acute respiratory syndrome coronavirus 2 (SARS-CoV-2) has concluded, diagnostic assays remain in demand to monitor SARS-CoV-2 at the individual patient level, regionally, and nationally, as well as to remain an infectious disease preparedness instrument to monitor any new SARS-CoV-2 dissemination across borders using population and wastewater surveillance. The anticipation by WHO and central health care policy entities such as the Center for Disease Control, EMA, and multiple national health authorities is that SARS-CoV-2 will reside as an endemic respiratory disease for years to come. The key strategic consideration is hence shifting from combating a pandemic situation with a high number of patients to instead allowing precise diagnostics of suspected patients with the intention of correct management in a low-prevalence setting.

## INTRODUCTION

The coronavirus disease 2019 (COVID-19) pandemic of the severe acute respiratory syndrome coronavirus 2 (SARS-CoV-2) continues to have a significant impact on global public health, economies, and societies. Through the 2-year initial phase of the pandemic, accurate and timely diagnosis has been crucial for disease control, prevention, and monitoring. As the pandemic phase has concluded, SARS-CoV-2 diagnostic assays remain in demand to monitor SARS-CoV-2 at the individual patient level to provide an infectious disease preparedness instrument using population and wastewater surveillance to monitor any emerging SARS-CoV-2 variants spreading across borders (1–5). Also discriminating SARS-CoV-2 alone or in the case of co-infections (6, 7) from other respiratory viruses in patients can help reduce the burden on healthcare systems and provide clinically relevant management of patients.

The pandemic created an urgent need for accurate and reliable diagnostic assays. Time pressure on the other hand forced regulatory agencies around the world to expedite approval processes for SARS-CoV-2 diagnostic assays under Emergency Use Authorization (EUA) (8–10). This accelerated procedure led to the exemption of the typical rigorous and stringent regulatory authorization approval process for diagnostics assays. As the pandemic waned, a return to the systematic standardized validation framework allowed catching up to ensure the quality of commercial SARS-CoV-2 diagnostic assays earlier EUA approved by the Food and Drug Administration and the European Medicines Agency (EMA). Here, assay validation with clinical samples is recommended to evaluate clinical accuracy. With this objective, the VALCOR (VALidation of SARS-CORona Virus-2 assays) protocol was developed to validate SARS-CoV-2 assays in a clinical framework (11), as previously demonstrated with the validation of the Aptima SARS-CoV-2 assay (Hologic, MA, USA) (12).

Synthetic and analytical SARS-CoV-2 RNA sequences have been generated as reference materials (RMs, also called standards), for quantification, calibration, quality control, and validation purposes of SARS-CoV-2 assays under development or already on the market. To our knowledge, there have been no reports regarding the alignment of SARS-CoV-2 assays available on the market with the existing SARS-CoV-2 RMs in terms of target genes and sequences of primers and probes used to validate both the clinical accuracy and the analytical limit of detection (LOD) of SARS-CoV-2 assays.

In this study, we validate the clinical accuracy of three Seegene Allplex SARS-CoV-2 assays (index assays) using clinical samples collected according to the VALCOR protocol (11). The three Seegene Allplex SARS-CoV-2 assays are Allplex SARS-CoV-2 (abbreviated as Allplex-SC2), Allplex SARS-CoV-2 Fast PCR (abbreviated as Allplex-SC2Fast) and the combi assay co-detection Influenza A and B along with respiratory syncytial virus, and the Allplex SARS-CoV-2/FluA/FluB/respiratory syncytial virus (RSV) (abbreviated as Allplex-SC2FabR). As a comparator, we employed the TaqPath COVID-19 assay (ThermoFisher Scientific, MA, USA). The TaqPath COVID-19 assay was previously validated in-house in the National Reference Laboratory for Respiratory Pathogens, University Hospitals Leuven (UZ Leuven), Belgium. The clinical performance of the three index assays was assessed as LOD in clinical samples of which a dilution series was prepared with 1:50 dilution as the maximum dilution. The analytical LOD of the three index assays and the comparator assay was assessed using two SARS-CoV-2 reference materials (RMs) (i.e., EURM-019 and RGTM 10169) that consist of three synthetic SARS-CoV-2 RNA fragments. Finally, the sequence concordance of target genes in the index assays, the comparator assay, and the RMs was assessed.

## MATERIALS AND METHODS

The full protocol of VALCOR and the composition of the VALCOR samples’ panel were described previously (11). In brief, three types of samples were investigated. (i) Clinical accuracy of index assay was assessed with 180 undiluted clinical samples (90 SARS-CoV-2 positives and 90 SARS-CoV-2 negatives) collected in the routine testing from the National Reference Laboratory for Respiratory Pathogens, Department of Laboratory Medicine (UZ Leuven, Belgium). The samples were collected while the Wuhan-Hu-1 strain was dominant and before the emergence of the Alpha SARS-CoV-2 variant. (ii) Forty dilutions of clinical SARS-CoV-2-positive samples (with a dilution matrix of 1:2, 1:10, 1:20, and 1:50) were used to assess the clinical LOD of the index assays and the comparator by constructing a standard curve with an RM. (iii) The analytical LOD of the index assays and the comparator was assessed by constructing a standard curve with synthetic SARS-CoV-2 RMs that were manufactured by RM manufacturers in Europe [Joint Research Centre (JRC), European Commission, Geel, Belgium] and in the USA [National Institute of Standards and Technology (NIST), Gaithersburg, USA). Additionally, sequences of target genes in the index assays, the comparator assay, and the RMs were assessed.

### Testing of samples with the index assays

Testing of the VALCOR panel with the three Seegene Allplex SARS-CoV-2 assays was carried out at the Laboratory of Molecular Diagnostics (GLMD–ZOL, Genk, Belgium). Nucleic acid extraction, based on magnetic beads, was performed on the Seegene STARlet extraction platform using 300 µL of input material (i.e., sample), resulting in a 100 µL eluate. Reverse transcriptase PCR (RT-PCR) and PCR were performed according to the manufacturer’s specifications for the Allplex-SC2, Allplex-SC2Fast, and Allplex-SC2FabR assays, respectively. Seegene Viewer analysis software was used for auto-interpretation of the results from the three Allplex assays, providing a binary outcome (i.e., presence or absence of SARS-CoV-2) along with Ct values. Viral load (VL), expressed in copies/mL, was estimated using a standard curve. Retesting of samples was carried out in case a sample tested positive for SARS-CoV-2 while its internal control was negative.

All three index assays are multiplex RT-PCR assays detecting the common SARS-CoV-2 target E, N, RdRP, and S genes (Table 1) (13): the Allplex-SC2 assay targets sequences within the E, RdRP/S, and N genes with the RdRP and S genes detected in the same fluorophore channel and using an exogenous internal control; the Allplex-SC2Fast assay targets the E, N, and RdRP genes and has an endogenous internal control; and the Allplex-SC2FabR assay detects N, RdRP, and S genes, has one exogenous and one endogenous internal control, and detects three additional pathogens, besides SARS-CoV-2. In the Allplex assays, a sample is considered positive if it detects at least one of the target genes of SARS-CoV-2 and the internal control is positive. Conversely, a sample is considered negative if all the target genes of SARS-CoV-2 are not detected, while the internal control is positive. According to the manufacturer’s specifications, all three Allplex assays can detect SARS-CoV-2 particles from nasopharyngeal swabs, saliva swabs, and combo swabs (nasal + oral swabs).

**TABLE 1:** SARS-CoV-2 genes of the targeted sequence of comparator, index assays, and three SARS-CoV-2 RMs

	SARS-CoV-2 gene of targeted sequence						Exogenous internal control
	E	N	RdRP	ORF1ab^*a*^	S	Endogenous internal control	
Assay							
TaqPath COVID-19		×		×	×		×
Allplex-SC2	×	×	×		×		×
Allplex-SC2Fast	×	×	×			×	
Allplex-SC2FabR		×	×		×	×	×
SARS-CoV-2 RMs							
EURM-019 (JRC)	×	×	×		×		
RGTM 10169-1 (NIST)	×	×					
RGTM 10169-2 (NIST)	×	×		×			

^*a*^ The ORF1ab gene includes the RdRP gene coding region.

### Testing of samples with the initial panel testing and a comparator assay

VALCOR samples were tested with the initial panel at UZ Leuven using the TaqPath COVID-19 assay as a comparator (11, 12). The initial panel testing was performed, between 1 April 2020 and 15 January 2021. Nucleic acids were extracted using the MagMax Viral Pathogen II extraction kit (ThermoFisher Scientific) on the KingFisher High Throughput platform. The target genes of Taqpath COVID-19 are N gene, ORF1ab gene, and S gene. The results were reported as log copies/mL based on the quantification cycle (Cq, also called Ct) value of the N gene. Detection of two or more target genes was considered a positive result. Samples were reported negative when all viral gene targets were Cq > 37 and the MS2 internal control was Cq < 27.5. VL was estimated using a standard curve and semi-quantitatively reported (14). In case of discordance between the initial panel testing and the TaqPath COVID-19 assay, a third assay, either Abbott Alinity *m* system (IL, USA), Aptima SARS-CoV-2 assay (Hologic Panther system, MA, USA) (12), or Xpert Xpress SARS-CoV-2 assay (Cepheid GeneXpert system, CA, USA), was deployed as an adjudicator.

### Testing of SARS-CoV-2 reference materials

**Fig 1:**
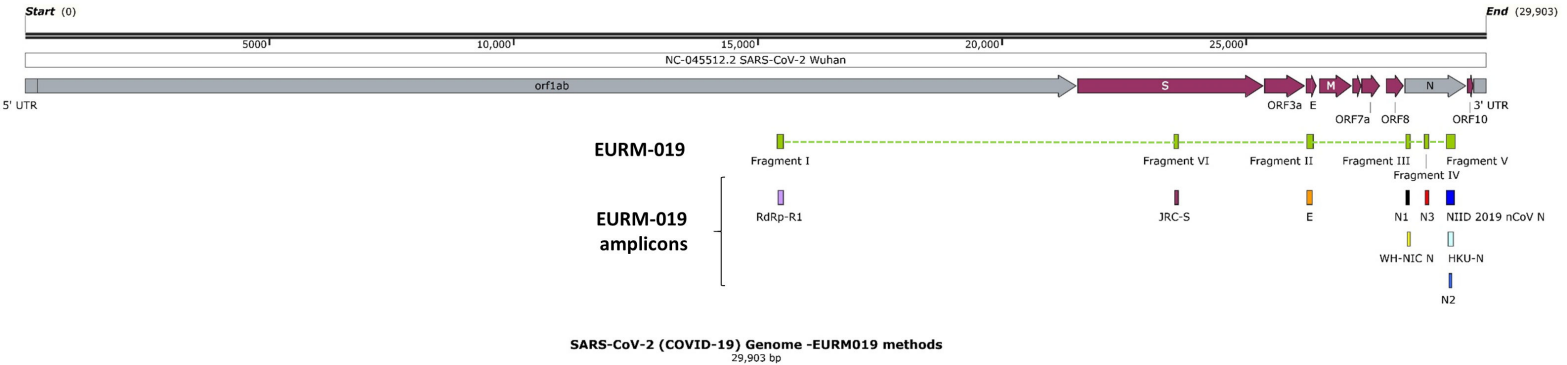
Schematic of EURM-019 synthetic ssRNA fragments, aligned against NC-045512.2 SARS-CoV-2 Wuhan strain. EURM-019 contains primers and probes of nine amplicons (i.e., RdRp-R1, JRC-S, E, N1, N3, NIID 2019 nCoV N, WH-NIC N, HKU-N, and N2).

**Fig 2:**
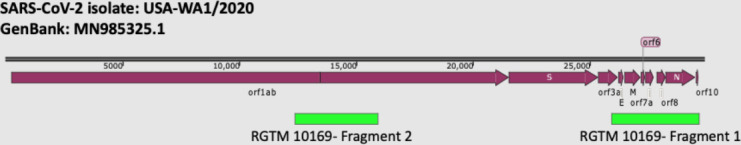
Schematic of RTGM 101069 synthetic RNA fragments.

EURM-019 (JRC, Geel, Belgium, https://crm.jrc.ec.europa.eu/p/EURM-019) is a 880-nt long *in vitro* transcribed synthetic single-stranded RNA (ssRNA) of the SARS-CoV-2 isolate with target regions from the E, N, RdRP, and S genes (GenBank accession ID: MN908947, Fig. 1). RGTM 10169 (NIST, Gaithersburg, USA, https://www.nist.gov/programs-projects/sars-cov-2-research-grade-test-material) is composed of two unique synthetic RNA sequences from the SARS-CoV-2 isolate (GenBank accession ID: MN985325.1), spiked with 5 ng/µL human Jurkat RNA (Fig. 2). The sequence fragments have a size of approximately 4 kb, covering positions 12,409–15,962 (named as RGTM 10169-1) and 25,949–29,698 (named as RGTM 10169-2). They contain the ORF1ab, E, and N genes (Table 1). For each of the three synthetic SARS-CoV-2 fragments, five dilutions were prepared with RNase-free water with poly-A-carrier RNA by JRC (Geel, Belgium, Table S1). The concentrations (or VLs) were measured using droplet digital RT-PCR (ddPCR) by JRC. The concentrations of the EURM-019 dilution series were 7.42 × 10^7^, 1.02 × 10^7^, 1.08 × 10^6^, 1.09 × 10^5^, and 1.30 × 10^4^ copies N3/mL (Table S1). The concentrations of the RGTM 10169-1 dilution series were 9.02 × 10^7^, 1.06 × 10^7^, 1.09 × 10^6^, 1.14 × 10^5^, and 1.00 × 10^4^ copies N3/mL (Table S1). The concentrations of the RGTM 10169-2 dilution series were 1.50 × 10^7^, 1.53 × 10^6^, 1.55 × 10^5^, 1.80 × 10^4^, and 9.00 × 10^2^ copies ORF1ab/mL (Table S1). The analytical LOD of an assay was determined by the detection of at least one of the target genes of the assay in the lowest dilution of these series. In addition, a standard curve was constructed with the dilution series of RMs, and based on the standard curve, the copies/mL values of samples were estimated. The LOD of a clinical diluted sample was determined by detecting at least one of the target genes of the assay in the lowest dilution level (i.e., 1:2, 1:10, 1:20, or 1:50; along with the respective copies/mL estimate).

### Statistical analyses

Concordance matrices were made with the paired data of the initial panel testing versus comparator, and comparator versus any index assay. The sensitivity of an index assay was defined as the proportion of true positives (TP) by the comparator and the index assay. The specificity of an index assay was defined as the proportion of true negatives (TN) by the comparator and the index assay to the number of SARS-CoV-2 negatives detected by the comparator. The overall concordance between the comparator and an index assay was defined as the proportion of the sum of TP and TN to the total number of SARS-CoV-2 positives and negatives in the study (*n* = 180). Exact binomial 95% confidence interval (CI) was calculated. Cohen’s kappa values (*κ*) were calculated (15) and categorized as (1.00 ≥ *κ* > 0.80) excellent; (0.80 ≥ *κ* > 0.60) good; (0.60 ≥ *κ* > 0.40) moderate; (0.40 ≥ *κ* > 0.20) fair; and (0.20 ≥ *κ* > 0.00) poor (16). Nonparametric Wilcoxon Signed Rank test was performed to determine if there was a statistically significant difference in the median-estimated VLs between the comparator and an index assay. Interquartile range 1 (Q1) and 3 (Q3) of estimated VLs were described. Statistical significance was set at 0.05. The Bland-Altman plots were visualized to describe a quantitative agreement between two VL measurements determined with two assays. An average VL difference substantially higher than zero indicates that the VL_TaqPath COVID-19_ tends to be higher than the VL_Seegene Allplex assay_ and vice versa. Statistical analyses were performed with STATA version 16 (College Station, TX, USA).

## RESULTS

### Panel samples versus TaqPath COVID-19 to establish the comparator

All undiluted negative panel samples tested negative with TaqPath COVID-19 (*n* = 90). Of the undiluted positive panel samples, 88 of 90 were positive using the comparator assay. The remaining two positive samples were further tested with an adjudicator assay. One sample was positive on two other platforms [Alinity *m* SARS-CoV-2 assay and Aptima SARS-CoV-2 assay (12)] and categorized as positive. The remaining sample tested negative on two other platforms (Alinity *m* SARS-CoV-2 assay and Xpert Xpress SARS-CoV-2 assay) and was categorized as negative. Overall, 89 non-diluted panel samples were positive for SARS-CoV-2 and 91 non-diluted panel samples were negative. Out of the 40 diluted clinical samples, four samples were negative with the TaqPath COVID-19 assay (two samples from 1:20 dilution level and two samples at 1:50 dilution level). The clinical LOD of the comparator assay was 5.51 × 10^3^ copies/mL.

### Clinical sensitivity, specificity, and LOD of the Seegene assays Allplex-SC2, Allplex-SC2Fast, and Allplex-SC2FabR compared to comparator

**Fig 3:**
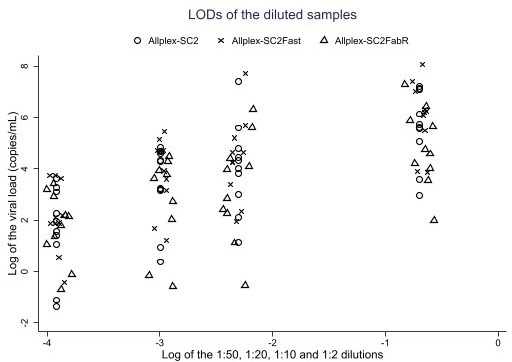
The limit of detections (i.e., 1:50, 1:20, 1:10, and 1:2 dilutions) and the viral loads (copies/mL, based on the N gene) of the clinical diluted samples for each Seegene Allplex assay, having the dilutions and the viral loads displayed in the logarithmic scale. Open circle: Allplex-SC2 assay. Cross: Allplex-SC2Fast. Open triangle: Allplex-SC2FabR.

All 89 undiluted positive samples tested positive with the Allplex-SC2, Allplex-SC2Fast, and Allplex-SC2FabR assays, resulting in a 100.0% sensitivity (95% CI = 95.9%–100.0%). Similarly, 89/91 undiluted negative panel samples were negative with the Allplex-SC2 and Allplex-SC2Fast assays, leading to a specificity of 97.8% (95% CI = 92.3%–99.7%). With the Allplex-SC2FabR assay, 91/91 undiluted samples were negative for SARS-CoV-2, leading to a specificity of 100.0% (95% CI = 95.9%–100.0%). The overall concordance of the comparator versus the Allplex-SC2 assay and the Allplex-SC2Fast assay was 98.9% (95% CI = 96.0%–99.9%) and the Cohen’s kappa (*κ*) was 0.978 (95% CI = 0.947–1.000). The overall concordance between the comparator and the Allplex-SC2FabR assay was 100.0% (95% CI = 98.0%–100.0%, one-sided) with a *κ* of 1.000 (95% CI = 1.000–1.000). The Allplex-SC2, Allplex-SC2Fast, and Allplex-SC2FabR assays could detect 40/40 diluted samples, up to the dilution level of 1:50. The clinical LOD was 2.61 × 10^2^ copies/mL for the Allplex-SC2 assay, 6.82 × 10^2^ copies/mL for the Allplex-SC2Fast assay and 3.67 × 10^2^ copies/mL for the Allplex-SC2FabR assay (Fig. 3).

### Target gene detection comparison between index assays and comparator

**Fig 4:**
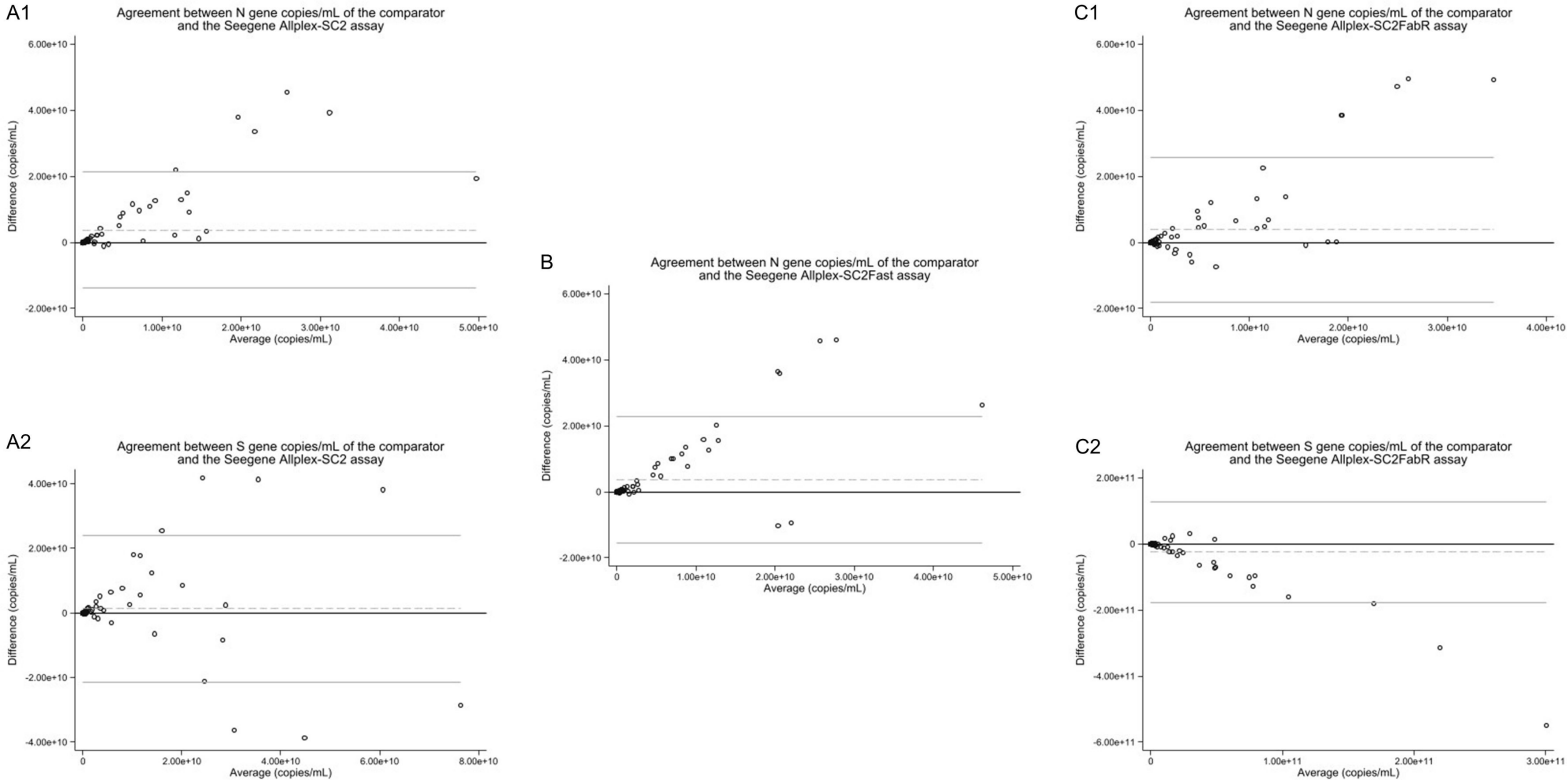
Bland Altman plots of the TaqPath COVID-19 assay, i.e., comparator assay, with (**A1 and A2**) the Seegene Allplex-SC2 assay (respectively, the N gene and the S gene), (**B**) the Seegene Allplex-SC2Fast assay and (**C1 and C2**) the Seegene Allplex-SC2FabR assay (respectively, the N gene and the S gene). The *x*-axis represents the average VL (copies/mL) of two assays, and the *y*-axis represents the VL difference (copies/mL) of two assays. The black horizontal line is the line of perfect agreement, i.e., zero in the VL differences on the *y*-axis. The dashed and gray horizontal line is the average of the VL differences. The upper and lower gray horizontal lines are, respectively, the upper and lower limits of agreement, i.e., ±1.96 times standard deviations of the pairwise VL differences.

The median estimated VLs for common target genes were lower with the index assays compared with the comparator, having the exception of the SARS-CoV-2 N gene with the Allplex-SC2Fast assay and the SARS-CoV-2 S gene with the Allplex-SC2FabR assay (Table 2). The median estimated VL for the N gene by the Allplex-SC2 assay was 7.32 × 10^7^ copies/mL (Q1–Q3: 2.23 × 10^6^–7.33 × 10^8^ copies/mL) compared to 2.23 × 10^8^ copies/mL (Q1–Q3: 1.61 × 10^7^–3.32 × 10^9^ copies/mL) by the comparator, having *P* < 0.0001. We observed similar results for the N gene by the Allplex-SC2FabR assay with a median estimated VL of 2.70 × 10^7^ copies/mL (Q1–Q3: 7.93 × 10^5^–1.36 × 10^9^ copies/mL), while the comparator shows 2.87 × 10^8^ copies/mL (Q1–Q3: 2.29 × 10^7^–3.96 × 10^9^ copies/mL) (*P* < 0.0001). The median estimated VL for the N gene by the Allplex-SC2Fast assay was 2.36 × 10^8^ copies/mL (Q1–Q3: 4.84 × 10^6^–1.47 × 10^9^ copies/mL), which is slightly higher compared to 2.23 × 10^8^ copies/mL (Q1–Q3: 1.61 × 10^7^–3.32 × 10^9^ copies/mL) by the comparator (*P* < 0.0001). When looking at the S gene, the median estimated VL by the Allplex-SC2 was 2.18 × 10^8^ copies/mL (Q1–Q3: 7.74 × 10^6^–2.82 × 10^9^ copies/mL), while 4.42 × 10^8^ copies/mL (Q1–Q3: 3.59 × 10^7^–5.30 × 10^9^ copies/mL) (*P* = 0.0001). With the Allplex-SC2FabR assay, the median estimated VL was 1.01 × 10^9^ copies/mL (Q1–Q3: 1.91 × 10^7^–1.47 × 10^10^ copies/mL), which is higher than the comparator (4.42 × 10^8^ copies/mL; Q1–Q3: 3.59 × 10^7^–5.29 × 10^9^ copies/mL; *z* = −3.607) (*P* = 0.0003). Figure 4 shows the Bland Altman plots of each comparison in Table 2.

**TABLE 2:** Distribution of the viral load (copies/mL) values for the common target genes in the samples that are commonly positive for TaqPath COVID-19 and the three Allplex assays^*a*^

Assay	Target gene	Median (copies/mL)	Q1–Q3 (copies/mL)	z score	*P*
TaqPath COVID-19 (C) Allplex-SC2 (I)	N (*n* = 88)	C: 2.23 × 10^8^	C: 1.61 × 10^7^–3.32 × 10^9^	6.599	<0.0001
		I: 7.32 × 10^7^	I: 2.23 × 10^6^–7.33 × 10^8^		
	S (*n* = 80)	C: 4.42 × 10^8^	C: 3.59 × 10^7^–5.30 × 10^9^	3.938	0.0001
		I: 2.18 × 10^8^	I: 7.74 × 10^6^–2.82 × 10^9^		
TaqPath COVID-19 (C) Allplex-SC2Fast (I)	N (*n* = 88)	C: 2.23 × 10^8^	C: 1.61 × 10^7^–3.32 × 10^9^	5.700	<0.0001
		I: 2.36 × 10^8^	I: 4.84 × 10^6^–1.47 × 10^9^		
TaqPath COVID-19 (C) Allplex-SC2FabR (I)	N (*n* = 84)	C: 2.87 × 10^8^	C: 2.29 × 10^7^–3.96 × 10^9^	4.491	<0.0001
		I: 2.70 × 10^7^	I: 7.93 × 10^5^–1.36 × 10^9^		
	S (*n* = 80)	C: 4.42 × 10^8^	C: 3.59 × 10^7^–5.29 × 10^9^	−3.607	0.0003
		I: 1.01 × 10^9^	I: 1.91 × 10^7^–1.47 × 10^10^		

^*a*^ C, comparator assay; I, index assay; Q1, interquartile range 1; and Q3, interquartile range 3.

### Analytical LOD assessment and sequence assessment of target genes in SARS-CoV-2 RM and Seegene Allplex SARS-CoV-2 assays

Out of the five dilutions of the three synthetic SARS-CoV-2 RNA reference materials (Table S1), both the Allplex-SC2 and Allplex-SC2Fast assays were able to successfully detect all of them (Table 3). The Allplex-SC2FabR assay showed positivity across all dilutions of RGTM 10169-1 and RGTM 10169-2 and in one dilution of EURM-019 (with a VL of 7.42 × 10^7^ copies/mL measured by ddPCR). The TaqPath COVID-19 assay could only detect the dilutions of RGTM 10169-1 (Table 3). The analytical LOD of the three Allplex assays was 9.00 × 10^2^ copies/mL, while the analytical LOD of the TaqPath COVID-19 assay was 1.00 × 10^4^ copies/mL. Table 3 shows that sequences of the target genes in the three index assays, the comparator assay, and the three RMs do not overlap completely. The positivity was driven by the N gene detection of RGTM 10169-1 in the TaqPath COVID-19 assay in comparison to the E, N, RdRP, or S gene in the Allplex-SC2 assay and the E, N, or RdRP gene in the Allplex-SC2Fast assay. The positivity in the Allplex-SC2FabR assay was driven by the N and RdRP genes, while it was not clear for the S gene. When looking from the RM perspective, EURM-019 was compatible with two Allplex assays: the Allplex-SC2 assay through the E, RdRP, and S genes; and the Allplex-SC2Fast assay through the E and RdRP genes, which means that the Allplex assays are probably based on the WHO-recommended methods. RGTM 10169-1 and RGTM 10169-2 were compatible with three Allplex assays, having different target genes in each synthetic RNA fragment. RGTM 10169-1 contained the N gene for the TaqPath COVID-19 assay and the three Allplex assays, and the E gene for two Allplex assays (i.e., Allplex-SC2 and Allplex-SC2Fast). RGTM 10169-2 contained the RdRP gene for the three Allplex assays.

**TABLE 3:** Analytical LOD (measured by ddPCR^*b*^) on the diluted synthetic RNA RMs for each target gene^*a*^

		TaqPath COVID-19	Allplex-SC2	Allplex-SC2Fast	Allplex-SC2FabR
		(N, ORF1ab, and S)	(E, N, and RdRP/S)	(E, N, and RdRP)	(N, RdRP, and S)
E	EURM-019 (JRC)	–	1.30 × 10^4^ copies N3/mL	1.30 × 10^4^ copies N3/mL	–
	RGTM 10169-1 (NIST)	–	1.00 × 10^4^ copies N3/mL	1.00 × 10^4^ copies N3/mL	–
	RGTM 10169-2 (NIST)	–	ND	ND	–
N	EURM-019 (JRC)	ND	ND	ND	ND
	RGTM 10169-1 (NIST)	1.00 × 10^4^ copies N3/mL	1.00 × 10^4^ copies N3/mL	1.00 × 10^4^ copies N3/mL	1.00 × 10^4^ copies N3/mL
	RGTM 10169-2 (NIST)	ND	ND	ND	ND
RdRP/ORF1ab^*c*^	EURM-019 (JRC)	ND	1.30 × 10^4^ copies N3/mL^*d*^	1.30 × 10^4^ copies N3/mL	ND
	RGTM 10169-1 (NIST)	ND	ND	ND	ND
	RGTM 10169-2 (NIST)	ND	9.00 × 10^2^ copies ORF1ab/mL^*d*^	9.00 × 10^2^ copies ORF1ab/mL	9.00 × 10^2^ copies ORF1ab/mL
S	EURM-019 (JRC)	ND	(1.30 × 10^4^ copies N3/mL)^*d*,*e*^	–	7.42 x 10^7^ copies N3/mL^*f*^
	RGTM 10169-1 (NIST)	ND	ND	–	ND
	RGTM 10169-2 (NIST)	ND	(9.00 × 10^2^ copies ORF1ab/mL) ^*d*,*e*^	–	ND

^*a*^ – indicates that the respective assay did not have the primers and probes of the target genes. ND, not detected, although the primers and probes of the assays were present in the RT-PCR.

^*b*^ ddPCR: droplet digital PCR.

^*c*^ The ORF1ab region includes the RdRP gene.

^*d*^ Primers and probes for RdRP/S gene of the Allplex-SC2 assay were in the same detection channel.

^*e*^ The detection of the RdRP and S gene were in the same channel; therefore, either the detection of the S gene might not be true.

^*f*^ Only one dilution (VL = 7.42 × 10^7^ copies N3/mL, measured by ddPCR) was positive.

## DISCUSSION

### Assay performance

In this study, we validated the assay performance of three Seegene Allplex SARS-CoV-2 assays using clinical SARS-CoV-2 samples within the VALCOR framework. The Allplex-SC2 and Allplex-SC2Fast assays had a sensitivity of 100.0% and a specificity of 97.8%, while the Allplex-SC2FabR assay had 100% sensitivity and specificity to the defined comparator, the TaqPath COVID-19 assay. The overall concordance for the three Allplex assays and the comparator was 98.9% for the Allplex-SC2 and Allplex-SC2Fast assays, and 100.0% for the Allplex-SC2FabR assay, having an excellent Cohen’s kappa of 0.978 and 1.000, respectively. A comparison with the performance of other validated SARS-CoV-2 assays, as reported in the literature, indicates that the Seegene Allplex SARS-CoV-2 assays perform at a level similar to the Aptima SARS-CoV-2 assay. The Aptima assay had also been validated using the VALCOR panel (12). In terms of sensitivity, the Seegene Allplex assays outperform the Abbott RealTime SARS-CoV-2 assay, achieving a sensitivity of 93% (17). It is worth noting that the Abbott RealTime assay, although achieving a lower sensitivity, was validated using a different set of clinical samples. The Abbott RealTime SARS-CoV-2 assay showed 100% specificity, which is similar to the Seegene Allplex-SC2FabR assay, while the Seegene Allplex-SC2 and Allplex-SC2Fast assays have a slightly lower specificity followed by the Aptima SARS-CoV-2 assay (96.7%) (12). The three Allplex assays were also compared with the TaqPath COVID-19 assay at the level of target genes. The N gene was common in the three Allplex assays and the TaqPath COVID-19 assay. The median estimated VL of the N and S genes in each Allplex assay is significantly different from the comparator, having a lower median estimated VL than the comparator. However, while the Allplex-SC2Fast and the Allplex-SC2FabR assay have a higher median estimated VL than the comparator, the direction of their *z* scores was opposite.

Time-to-result is crucial for any respiratory patient management, and the runtime of Allplex-SC2 and Allplex-SC2FabR assays was approximately 2 hours, while 1 hour for the Allplex-SC2Fast assay.

### Limit of detection

The clinical LOD was evaluated using clinical samples by the three Allplex SARS-CoV-2 assays, revealing a clinical LOD lower than the comparator assay. Similar findings were observed during the analytical LOD assessment, which utilized three SARS-CoV-2 RMs, and the three Allplex assays exhibited a slightly higher sensitivity than the TaqPath COVID-19 assay. Notably, the clinical LODs with the three Allplex assays were even marginally lower than the analytical LODs.

### Target gene sequences in RMs

Target genes of the three Seegene Allplex SARS-CoV-2 assays were detected in the SARS-CoV-2 RMs, fulfilling the minimum requirement of viral genes as included in the RMs. Nonetheless, the sequence of target genes was not consistent between these Allplex assays and the RMs since not all target genes could be detected at once in the RMs (Table 4). The TaqPath COVID-19 assay could detect fewer target genes of the three synthetic RNA fragments than expected (Table 4—“after sequence assessment” versus “before sequence assessment”). Possible explanations for this could include the (partial) absence of the targeted gene sequences in the RMs or an improper sequence alignment of the primers and probes used in the assays with the RM. As a secondary finding, RGTM 10169-2 contained fragments of the RdRP gene, which were not stated in the reference sequence. However, the detection of the RdRP and S genes was in the same fluorophore channel of the Allplex-SC2 assay, and hence specific detection of the S gene cannot be determined.

**TABLE 4:** Target genes of the TaqPath COVID-19 assay, the three Allplex assays, and the three synthetic RNA RMs: before and after sequence assessment of target genes

	Target gene			
	TaqPath COVID-19	Allplex-SC2	Allplex-SC2Fast	Allplex-SC2FabR
Before sequence assessment				
EURM-019 (JRC)	N, S	E, N, RdRP, S	E, N, RdRP	N, RdRP, S
RGTM 10169-1 (NIST)	N, ORF1ab	E, N	E, N	N
RGTM 10169-2 (NIST)	N, ORF1ab	E, N	E, N	N
After sequence assessment				
EURM-019 (JRC)	–^*c*^	E, RdRP, S	E, RdRP	S^*a*^
RGTM 10169-1 (NIST)	N	E, N	E, N	N
RGTM 10169-2 (NIST)	–^*c*^	RdRP (S^*b*^)	RdRP	RdRP

^*a*^ Undetermined, as only one dilution (VL = 7.42 x 10^7^ copies N3/mL, measured by ddPCR) was positive.

^*b*^ The detection of the RdRP and S genes was in the same fluorophore channel, which implies that the detection of the S gene might not be true.

^*c*^ Target gene(s) not detected.

### Limitations

Throughout the pandemic, clinical samples were tested using available but not necessarily the same assays on different platforms, and assays were used in parallel to address the limited diagnostic capacity experienced across the globe. Also, no SARS-CoV-2 standard comparator was available at that time of study. The choice of the SARS-CoV-2 assay of UZ Leuven as a comparator also represents a limitation because the VALCOR panel relies on residual clinical samples, which is a challenge to scale up. The initial sample volume was relatively low, approximately ranging from 2 to 3 mL, again limiting the number of panels available. Given that the VALCOR panel of this study was collated during the first peak of the COVID-19 epidemic in Belgium, at a time when the Wuhan-Hu-1 strain was dominant and prior to the emergence of the Alpha SARS-CoV-2 variant, it is possible that there may be a bias in terms of SARS-CoV-2 variants. Furthermore, a limitation of the VALCOR panel is the absence of other viruses within its composition. This omission missed the opportunity for the assessment of cross-reactivity, for instance, FluA/FluB/RSV, in terms of specificity. Incorporation of a variety of SARS-CoV-2 variants into future VALCOR panels will enhance the generalization of SARS-CoV-2 assays’ validation, similar to the built-in heterogeneity in the four VALGENT [VALidation of Human Papilloma Virus (HPV) GENotyping Test] panels (18). VALCOR is a framework for validating SARS-CoV-2 virus assays and is inspired by the principles of VALGENT, which is a globally recognized forum for HPV assay comparison and validation (19). Also, due to the lack of primer sequences from commercially available SARS-CoV-2 assays, sequence alignment analysis (i.e., TaqPath COVID-19 assay) could not be performed to inspect the compatibility between the assays and the circulating variants. When looking at the results of the *in silico* alignment prediction with the EURM-019 RM and the recent SARS-CoV-2 variants against the reference strain Wuhan-Hu-1, one amplicon [i.e., WH-NIC N amplicon (Fig. 1)] is not predicted to amplify the concerned sequence in the recent SARS-CoV-2 variants. Out of the nine amplicons in the EURM-019, four amplicons contain at least one position in the sequence where the nucleotide is not aligned with the Wuhan-Hu-1 strain. However, the actual alignment and amplification depend on the forward primer, the reverse primer, the probe, and their physical properties as a whole (such as sequence and GC content). The correlation to clinical outcome is a general limitation when characterizing the analytical LOD of any diagnostic assays, and especially so for SARS-CoV-2 given the number of co-morbidities influencing whether a person becomes severely sick or not by SARS-CoV-2.

### Perspectives of SARS-CoV-2 assay validations

In the pandemic phase, all available molecular technologies were brought into play to address the immediate demands of healthcare services worldwide. In this context, real-time reverse transcriptase polymerase chain reaction has served as the workhorse and gold standard technology for SARS-CoV-2 assays, enabling the quantification of SARS-CoV-2 copy numbers (20, 21). In comparison, ddPCR has a higher analytical sensitivity than the real-time RT-PCR technology (22) also with quantification and absolute quantification of SARS-CoV-2 copy numbers. Another assay technology that is used in rapid or point-of-care detection assay is the CRISPR (clustered regularly interspaced short palindromic repeats) technology, which can be combined with reverse transcriptase loop-mediated amplification or recombinase polymerase amplification (23–26). The turnaround time of such a CRISPR-based assay is around 50 min, which is nearly similar to the Seegene Allplex SARS-CoV-2 Fast PCR assay (27). High sensitivity and a fast turnaround time of SARS-CoV-2 assays are crucial in the early detection of outbreaks in the population and their surrounding environment. Wastewater surveillance is gaining significance in the early detection of outbreaks, offering passive surveillance that has the potential to mitigate population incentives and mobility activities (28–30). A duplex real-time RT-PCR SARS-CoV-2 assay based on two ultra-conserved SARS-CoV-2 sequences has been reported to detect all variants (including the Omicron variant) in clinical and wastewater samples (31, 32). This duplex assay has demonstrated similar assay performance to that of the Seegene Allplex SARS-CoV-2 assay.

As the COVID-19 pandemic has passed, the anticipation by WHO and central health care policy entities such as Centers for Disease Control, EMA, and multiple national health authorities is that SARS-CoV-2 will reside as an endemic respiratory disease, and thus a return to standard operating practice with respect to evidence-based medicine is called for.

For clinical diagnostic purposes, combined assays detecting SARS-CoV-2, influenza virus, and/or RSV (6, 7), such as the Allplex-SC2FabR assay can allow precise diagnostics of suspected patients with the intention of correct management in a low-prevalence setting. Confounding the clinical presentation of COVID-19 are other respiratory virus infections such as influenza and RSV, which play a role in screening and diagnosing primary and co-infections, including identification of the pathogen in question (33, 34).

### Conclusion

All three Seegene Allplex SARS-CoV-2 assays demonstrated very high clinical accuracy, with 100% sensitivity and specificity ranging from 98% to 100%. The Allplex assays remained positive up to dilution 1:50 of clinical samples, which was not the case with the TaqPath COVID-19 assay. The analytical LODs (measured by ddPCR and using reference materials) were lower for the three Allplex assays compared to the TaqPath COVID-19 assay. The clinical LOD was slightly lower when compared to the analytical LOD using the Allplex assays. The target gene assessment revealed that target gene sequences of the Seegene Allplex SARS-CoV-2 assays and the SARS-CoV-2 reference materials were not completely overlapping.

## Data Availability

Data sets generated by VALCOR were stored locally and securely at Sciensano. Anonymized data can be made available upon request to the corresponding author on a case-by-case basis pending approval from the information security coordinator at Sciensano.

## SUPPLEMENTAL MATERIAL

Table S1 spectrum.02397-23-s0001.docx SARS-CoV-2 reference material dilution series.

ASM does not own the copyrights to Supplemental Material that may be linked to, or accessed through, an article. The authors have granted ASM a non-exclusive, world-wide license to publish the Supplemental Material files. Please contact the corresponding author directly for reuse.
