# Supplementary material for: Assessment of the clinical and analytical performance of three Seegene Allplex SARS-CoV-2 assays within the VALCOR framework
Source: Microbiol Spectr. 2024 Jan 8;12(2):e02397-23. doi: 10.1128/spectrum.02397-23 (PMC10846132; doi:10.1128/spectrum.02397-23)
Supplement: Table S1 — SARS-CoV-2 reference material dilution series. [file spectrum.02397-23-s0001.docx]

**Assessment of the clinical and analytical performance of three Seegene Allplex SARS-CoV-2 assays within the VALCOR framework**

Pui Yan Jenny Chung^1^, Sharonjit K. Dhillon^1^, Cindy Simoens^1^, Lize Cuypers^2,3^, Lies Laenen^2,3^, Jesper Bonde^4^, Philippe Corbisier^5^, Gerhard Buttinger^5^, Clementina E. Cocuzza^6^, Steven Van Gucht^7^, Marc Van Ranst^8^, Marc Arbyn^1,9^*

^1^ Unit Cancer Epidemiology, Belgian Cancer Centre, Sciensano, Brussels, Belgium

^2^ National Reference Centre for Respiratory Pathogens, Department of Laboratory Medicine, University Hospitals Leuven, Leuven, Belgium

^3^ Laboratory of Clinical Microbiology, Department of Microbiology, Immunology and Transplantation, KU Leuven, Leuven, Belgium

^4^ Molecular Pathology Laboratory, Department of Pathology, AHH-Hvidovre Hospital, Copenhagen University Hospital , Copenhagen, Denmark

^5^ European Commission, Joint Research Centre, Geel, Belgium

^6^ Laboratory of Clinical Microbiology and Virology, Department of Medicine and Surgery, University of Milano – Bicocca, Monza, Italy

^7^ Service of Viral Diseases, Sciensano, Brussels, Belgium

^8^ Laboratory of Clinical and Epidemiological Virology, Department of Microbiology, Immunology and Transplantation, Rega Institute for Medical Research, KU Leuven, Leuven, Belgium

^9^ Department of Human Structure and Repair, Faculty of Medicine and Health Sciences, University of Ghent, Ghent, Belgium

*Corresponding author

**Correspondence:** Marc Arbyn, Unit Cancer Epidemiology, Belgian Cancer Centre, Sciensano,

J. Wytsmanstreet 14, B1050 Brussels, Belgium.

E-mail: marc.arbyn@sciensano.be

**Supplementary Tables**

**Supplementary Table 1. SARS-CoV-2 reference materials dilution series**

| Step | Dilution | µL | Solution  (N3 copies/mL) | Dilution factor | Solution (µL) | Diluent* (µL) | Total volume (µL) | Nominal  (copies N3/mL) | Measured by ddPCR | | RSD |
| --- | --- | --- | --- | --- | --- | --- | --- | --- | --- | --- | --- |
|  |  |  |  |  |  |  |  |  | **copies N3/mL** | **stdev** |  |
| *EURM-019* | | | | | | | | | |  |  |
| 1 |  | 100 | 7.00 x 10^10^ | 70 | 10 | 690 | 700 | 1.00 x 10^9^ |  |  |  |
| 2 | Dilution 1 | 700 | 1.00 x 10^9^ | 10 | 250 | 2250 | 2500 | 1.00 x 10^8^ | 7.42 x 10^7^ | 4.64 x 10^6^ | 6% |
| 3 | Dilution 2 | 2500 | 1.00 x 10^8^ | 10 | 250 | 2250 | 2500 | 1.00 x 10^7^ | 1.02 x 10^7^ | 4.64 x 10^5^ | 5% |
| 4 | Dilution 3 | 2500 | 1.00 x 10^7^ | 10 | 250 | 2250 | 2500 | 1.00 x 10^6^ | 1.08 x 10^6^ | 1.70 x 10^4^ | 2% |
| 5 | Dilution 4 | 2500 | 1.00 x 10^6^ | 10 | 250 | 2250 | 2500 | 1.00 x 10^5^ | 1.09 x 10^5^ | 7.00 x 10^3^ | 6% |
| 6 | Dilution 5 | 2500 | 1.00 x 10^5^ | 10 | 250 | 2250 | 2500 | 1.00 x 10^4^ | 1.30 x 10^4^ | 2.00 x 10^3^ | 15% |
| *RGTM 10169-1* | | | | | | | | | | | |
| 1 | Dilution 1 | 110 | 5.00 x 10^9^ | 50 | 50 | 2450 | 2500 | 1.00 x 10^8^ | 9.02 x 10^7^ | 1.61 x 10^7^ | 18% |
| 2 | Dilution 2 | 2500 | 1.00 x 10^8^ | 10 | 250 | 2250 | 2500 | 1.00 x 10^7^ | 1.06 x 10^7^ | 1.06 x 10^6^ | 10% |
| 3 | Dilution 3 | 2500 | 1.00 x 10^7^ | 10 | 250 | 2250 | 2500 | 1.00 x 10^6^ | 1.09 x 10^6^ | 1.17 x 10^5^ | 11% |
| 4 | Dilution 4 | 2500 | 1.00 x 10^6^ | 10 | 250 | 2250 | 2500 | 1.00 x 10^5^ | 1.14 x 10^5^ | 1.40 x 10^4^ | 12% |
| 5 | Dilution 5 | 2500 | 1.00 x 10^5^ | 10 | 250 | 2250 | 2500 | 1.00 x 10^4^ | 1.00 x 10^4^ | 3.00 x 10^3^ | 30% |
| *RGTM 10169-2* | | | | | | | | | | | |
| 1 | Dilution 1 | 110 | 4.10 x 10^8^ | 50 | 50 | 2450 | 2500 | 8.20 x 10^6^ | 1.50 x 10^7^ | 1.58 x 10^6^ | 11% |
| 2 | Dilution 2 | 2500 | 8.20 x 10^6^ | 10 | 250 | 2250 | 2500 | 8.20 x 10^5^ | 1.53 x 10^6^ | 1.75 x 10^5^ | 11% |
| 3 | Dilution 3 | 2500 | 8.20 x 10^5^ | 10 | 250 | 2250 | 2500 | 8.20 x 10^4^ | 1.55 x 10^5^ | 1.80 x 10^4^ | 12% |
| 4 | Dilution 4 | 2500 | 8.20 x 10^4^ | 10 | 250 | 2250 | 2500 | 8.20 x 10^3^ | 1.80 x 10^4^ | 4.00 x 10^3^ | 22% |
| 5 | Dilution 5 | 2500 | 8.20 x 10^3^ | 10 | 250 | 2250 | 2500 | 8.20 x 10^2^ | 9.00 x 10^2^ | 5.00 x 10^2^ | 56% |

*The diluant was RNAse free water (molecular biology grade) including poly-A carrier RNA (conc. = 10 ng/µL) with carrier RNA from AppliedBiosystems Thermofisher Scientific (conc. = 1 mg/µL). RSD: relative standard deviation.
